# Characterizing 24-Hour Skeletal Muscle Gene Expression Alongside Metabolic and Endocrine Responses Under Diurnal Conditions

**DOI:** 10.1210/clinem/dgae350

**Published:** 2024-05-23

**Authors:** Harry A Smith, Iain Templeman, Max Davis, Tommy Slater, David J Clayton, Ian Varley, Lewis J James, Benita Middleton, Jonathan D Johnston, Leonidas G Karagounis, Kostas Tsintzas, Dylan Thompson, Javier T Gonzalez, Jean-Philippe Walhin, James A Betts

**Affiliations:** Centre for Nutrition, Exercise and Metabolism, Department for Health, University of Bath, Bath, UK, BA2 7AY; Centre for Nutrition, Exercise and Metabolism, Department for Health, University of Bath, Bath, UK, BA2 7AY; Centre for Nutrition, Exercise and Metabolism, Department for Health, University of Bath, Bath, UK, BA2 7AY; Musculoskeletal Physiology Research Group, Sport, Health and Performance Enhancement Research Centre, School of Science and Technology, Nottingham Trent University, Nottingham, UK, NG1 4FQ; Musculoskeletal Physiology Research Group, Sport, Health and Performance Enhancement Research Centre, School of Science and Technology, Nottingham Trent University, Nottingham, UK, NG1 4FQ; Musculoskeletal Physiology Research Group, Sport, Health and Performance Enhancement Research Centre, School of Science and Technology, Nottingham Trent University, Nottingham, UK, NG1 4FQ; National Centre for Sport and Exercise Medicine, School of Sport, Exercise and Health Sciences, Loughborough University, Loughborough, UK, LE11 3TU; Section of Chronobiology, School of Biosciences, Faculty of Health and Medical Sciences, University of Surrey, Guildford, UK, GU2 7XH; Section of Chronobiology, School of Biosciences, Faculty of Health and Medical Sciences, University of Surrey, Guildford, UK, GU2 7XH; Institute of Social and Preventive Medicine, University of Bern, 3012 Bern, Switzerland; Mary MacKillop Institute for Health Research (MMIHR), Australian Catholic University (ACU), Melbourne, VIC 3000, Australia; MRC Versus Arthritis Centre for Musculoskeletal Ageing Research, School of Life Sciences, University of Nottingham, Queen's Medical Centre, Nottingham, UK, NG7 2UH; Centre for Nutrition, Exercise and Metabolism, Department for Health, University of Bath, Bath, UK, BA2 7AY; Centre for Nutrition, Exercise and Metabolism, Department for Health, University of Bath, Bath, UK, BA2 7AY; Centre for Nutrition, Exercise and Metabolism, Department for Health, University of Bath, Bath, UK, BA2 7AY; Centre for Nutrition, Exercise and Metabolism, Department for Health, University of Bath, Bath, UK, BA2 7AY

**Keywords:** skeletal muscle, gene expression, circadian rhythms, diurnal, glucose, lipids

## Abstract

**Context:**

Skeletal muscle plays a central role in the storage, synthesis, and breakdown of nutrients, yet little research has explored temporal responses of this human tissue, especially with concurrent measures of systemic biomarkers of metabolism.

**Objective:**

To characterize temporal profiles in skeletal muscle expression of genes involved in carbohydrate metabolism, lipid metabolism, circadian clocks, and autophagy and descriptively relate them to systemic metabolites and hormones during a controlled laboratory protocol.

**Methods:**

Ten healthy adults (9M/1F, [mean ± SD] age 30 ± 10 years; BMI 24.1 ± 2.7 kg·m^−2^) rested in the laboratory for 37 hours with all data collected during the final 24 hours (08:00–08:00 hours). Participants ingested hourly isocaloric liquid meal replacements alongside appetite assessments during waking before a sleep opportunity from 22:00 to 07:00 hours. Blood samples were collected hourly for endocrine and metabolite analyses, with muscle biopsies occurring every 4 hours from 12:00 to 08:00 hours the following day to quantify gene expression.

**Results:**

Plasma insulin displayed diurnal rhythmicity peaking at 18:04 hours. Expression of skeletal muscle genes involved in carbohydrate metabolism (*Name*, Acrophase [hours]: *GLUT4*, 14:40; *PPARGC1A*, 16:13; *HK2*, 18:24) and lipid metabolism (*FABP3*, 12:37; *PDK4*, 05:30; *CPT1B*, 12:58) displayed 24-hour rhythmicity that reflected the temporal rhythm of insulin. Equally, circulating glucose (00:19 hours), nonesterified fatty acids (04:56), glycerol (04:32), triglyceride (23:14), urea (00:46), C-terminal telopeptide (05:07), and cortisol (22:50) concentrations also all displayed diurnal rhythmicity.

**Conclusion:**

Diurnal rhythms were present in human skeletal muscle gene expression as well systemic metabolites and hormones under controlled diurnal conditions. The temporal patterns of genes relating to carbohydrate and lipid metabolism alongside circulating insulin are consistent with diurnal rhythms being driven in part by the diurnal influence of cyclic feeding and fasting.

The human circadian system consists of both central (suprachiasmatic nuclei; SCN) and peripheral (eg, muscle, liver, adipose) clocks. These allow for temporal coordination of core physiological processes with cyclic environmental and behavioral events such as light-dark, waking-sleeping, activity-rest, and feeding-fasting.

Daily variations in nutrient metabolism are apparent; glucose tolerance is generally lower in the evening than in the morning, whereas lipid metabolism favors progressively elevated circulating lipids later in the day and into the night (1-10). Diurnal regulation of insulin secretion/clearance and sensitivity drives rhythmicity in both carbohydrate and lipid metabolism (11), with lipid metabolism further dictated by rhythmic intestinal triglyceride absorption, lipoprotein lipase (LPL) activity, mitochondrial oxidative capacity, and very low-density lipoprotein (VLDL) secretion (7, 9, 10, 12-17). Equally, circulating catabolic and anabolic markers, such as cortisol and testosterone, also exhibit rhythmicity across the day, both peaking in the morning (18, 19). Daily variation in these hormones may contribute to day-night rhythms in muscle protein metabolism (20) but may also further contribute to observed daily profiles in circulating glucose and lipids (21-23). Despite possible interactions between these rhythms, there are limited human data regarding temporal relationships between metabolic and endocrine markers of carbohydrate, lipid, and protein metabolism.

Skeletal muscle displays robust rhythmicity in transcriptomic regulation of the circadian clock, as well as carbohydrate, lipid, and protein metabolism; this may influence the central role of this tissue in the storage, synthesis, and breakdown of nutrients (13, 24-28). Specifically, skeletal muscle is an important storage site for glucose (glycogen) (29, 30) and lipids (intramyocellular lipids) (27, 31), and is also the primary store of protein within the human body (32-35). The ability to readily dispose and mobilize these nutrients from this tissue is an important determinant of insulin sensitivity and therefore metabolic health (27, 31, 36-38). Furthermore, autophagy is a central process that regulates skeletal muscle protein turnover, as well as glucose and lipid metabolism, and it responds to a variety of stimuli, including nutrient deprivation and amino acid starvation (39, 40). However, no studies have explored molecular regulation of this process within skeletal muscle across a 24-hour period. Considering the importance of the skeletal muscle in facilitating the response to nutrient availability, it is remarkable that no studies to date have assessed rhythmicity in the molecular regulation of skeletal muscle metabolism alongside circulating metabolites and hormones involved in carbohydrate and lipid metabolism and bone resorption.

Previous studies employing constant routine protocols to study daily variation in carbohydrate/lipid metabolism have provided valuable insight into endogenous circadian rhythmicity in the absence of behavioral rhythms. However, glucose and lipid metabolism are strongly modulated by diurnal behavioral factors, including fasting duration (41), physical activity/exercise (42, 43), sleep (44), and food/macronutrient intake/timing (45-48). During typical schedules, behavioral rhythms such as feeding-fasting are naturally aligned with cycles of light-dark and wake-sleep, such that the majority of daylight hours are spent in the postprandial state, with the longest period of fasting across 24-hour period occurring at night (49). Given the divergent responses of circulating insulin to feeding and fasting, alongside the potent entrainment effect of insulin upon circadian clocks, it is vital to study such metabolic rhythms in the context of these diurnal influences (50-52).

To enhance our knowledge of metabolic regulation across a 24-hour period of tightly controlled light-dark exposure and sleep-wake opportunity, it is now important to assess systemic hormonal and metabolite profiles alongside simultaneously collected skeletal muscle samples. To this end, the aim of this study was to characterize 24-hour rhythms in skeletal muscle expression of genes involved in nutrient metabolism and autophagy alongside systemic metabolites and hormones, during a semi-constant routine whereby feeding-fasting was aligned with light-dark exposure and wake-sleep opportunity.

## Methods

### Approach to the Research Question

Given the protracted nature of this study, a single-arm time-series design was deemed appropriate. Whereas constant routine studies eliminate the influence of diurnal factors, such as sleep-wake and fasting-feeding, the current study employed a semi-constant routine to study the diurnal influence of those factors. This protocol was characterized by designated wake and sleep opportunities that were aligned with feeding and fasting, respectively. Specifically, isocaloric snacks were ingested by participants every hour during waking hours to align feeding-fasting with wake-sleep and light-dark, respectively. Hourly feeds were prescribed to provide 6.66%·h^−1^ of estimated 24 hours resting metabolic rate (RMR) across the 15-hour waking period (ie, 08:00–22:00 hours), thus meeting individually-measured resting energy requirements and accounting for RMR as a driver of energy intake (53, 54). This model of continuous (hourly) feeding was selected to facilitate characterization of the underlying 24-hour fed-fast rhythm in the absence of the acute meal responses that would occur with any particular meal pattern. Nonetheless, the overall 24-hour pattern of nutrient availability with this model of continuous feeding is not dissimilar to that observed with a typical 3-square-meal pattern (even without snacking) since, even though nutrients are commonly ingested only periodically by most humans, there is a constant systemic appearance of nutrients from the gastrointestinal tract for the entirely of waking hours (49).

Hourly blood sampling was deemed both sufficient and feasible to detect diurnal rhythmicity in systemic hormones and metabolites (55, 56). Conversely, a different approach was required for muscle sampling due to the invasive nature of collecting these samples. Sampling at 4-hour intervals was deemed appropriate to assess rhythmic expression of metabolic genes in this tissue while also minimizing participant discomfort.

Transcriptomic data from the same participants have been reported previously in an untargeted analysis of rhythmicity (25). The aim of the current study was to analyze skeletal muscle RNA levels in a targeted number of metabolic genes in order to contrast with rhythms in circulating biomarkers. Plasma melatonin has also been reported previously and is included in the current manuscript to illustrate 24-hour profiles relative to diurnal melatonin and melatonin onset (24, 50). Likewise, cortisol from this protocol has also been reported previously at 4-hourly resolution aligned with muscle biopsy samples (24); updated biochemical analyses were therefore deemed necessary to increase resolution and capture the profile of cortisol prior to the first biopsy at midday (08:00–12:00 hours).

### Research Design

A time-series design was employed to investigate temporal rhythms in skeletal muscle gene expression relating to carbohydrate metabolism, lipid metabolism, circadian clocks, and autophagy, alongside plasma glucose, nonesterified fatty acids, insulin, glycerol, triglycerides, and C-terminal telopeptide (CTX), as well as serum cortisol and testosterone under conditions of semi-constant routine. Following a 7-day period of standardized wake-sleep, meal-timing, and light exposure (a typical living pattern for this population, thus serving to reduce between-participant variation in response to the semi-constant routine), participants underwent a 37-hour inpatient visit to the resting laboratory at the University of Bath. During the final 24 hours of this visit, participants had a designated sleeping opportunity (22:00−07:00 hours) and hourly isocaloric feedings during waking periods (08:00–22:00 hours) to preserve diurnal influences of sleep-wake and fasting-feeding. Hourly blood samples were collected throughout the day (while awake) and night (during sleep) for assessment of rhythms in the systemic concentrations of glucose, nonesterified fatty acids, and insulin, along with melatonin and cortisol to provide a validated internal phase marker. Skeletal muscle samples were collected every 4 hours from 12:00 hours for the remainder of the trial for assessment of RNA expression.

### Participants

Ten healthy participants (9 male; 1 female, Table 1), who maintained a typical sleep-wake cycle (ie, not of extreme chronotype and kept a consistent daily routine) and who did not perform shift work, were recruited and screened via local advertisement. Participant screening was undertaken through completion of a general health questionnaire and validated chronotype questionnaires to assess habitual sleep patterns and diurnal preferences (57-59). Participants were excluded from participation if they had a habitual sleep duration not within 6 to 9 hours per night and/or a Pittsburgh Sleep Quality Index > 5. With regards to shift work, exclusion criteria were in place for individuals who had participated in shift work or had traveled across more than 2 time zones within 3 weeks of the study. All volunteers were fully briefed on the requirements of the study prior to provision of written informed consent. Ethical approval for the experimental protocol was obtained from the Cornwall and Plymouth NHS research ethics committee (reference: 14/SW/0123). All procedures were performed in accordance with the Declaration of Helsinki.

**Table 1.** Participant characteristics of the study cohort

Characteristic	Mean ± SD
Age (y)	30 ± 10
Height (m)	1.81 ± 0.06
Body mass (kg)	78.7 ± 7.0
Body mass index (kg·m^−2^)	24.1 ± 2.7
Resting metabolic rate (kcal·day^−1^)	1724 ± 314
Midsleep time (hh:mm)*^a^*	03:42 ± 01:13
Horne-Östberg score	57 ± 11
Pittsburgh Sleep Quality Index	3 ± 2

Data are presented as mean ± SD.

^a^Determined from the Munich Chronotype Questionnaire (57).

### Pre-Experimental Standardization Week

Participants adhered to a strict routine of feeding and sleeping in the 7 days prior to entering the laboratory, waking between 06:00 and 07:00 hours and going to sleep between 22:00 and 23:00 hours, confirmed using time-stamped voicemail. The median (interquartile range) time that those voicemails were received were 06:53 hours (06:43-07:22) for waking and 22:45 hours (22:30–22:50) for lights out, respectively.

Upon waking, participants ensured at least 15 minutes of natural light exposure within 1.5 hours of waking, affirmed by wrist actigraphy using a light sensor, further confirming standardization of sleep-wake patterns (Actiwatch^TM^, Cambridge Neurotechnology; Cambridge, UK). Self-selected meals were scheduled at 08:00, 12:00 and 18:00 hours, with assigned snacking opportunities at 10:00, 15:00 and 20:00 hours. Participants also completed a weighed record of all food and fluid intake on the final 2 days of this 7-day standardization period and verified that they had consumed the reported meals and snacks at the prescribed times (Table 2).

**Table 2.** Dietary intake in the 48 hours prior to the laboratory visit

	Mean ± SD
Energy (kcal)	3002 ± 726
Carbohydrate (kcal)	1279 ± 357
Protein (kcal)	551 ± 235
Fat (kcal)	520 ± 176
Alcohol (kcal)	0 ± 0

Data are presented as mean ± SD.

### Experimental Protocol

**Figure 1.**
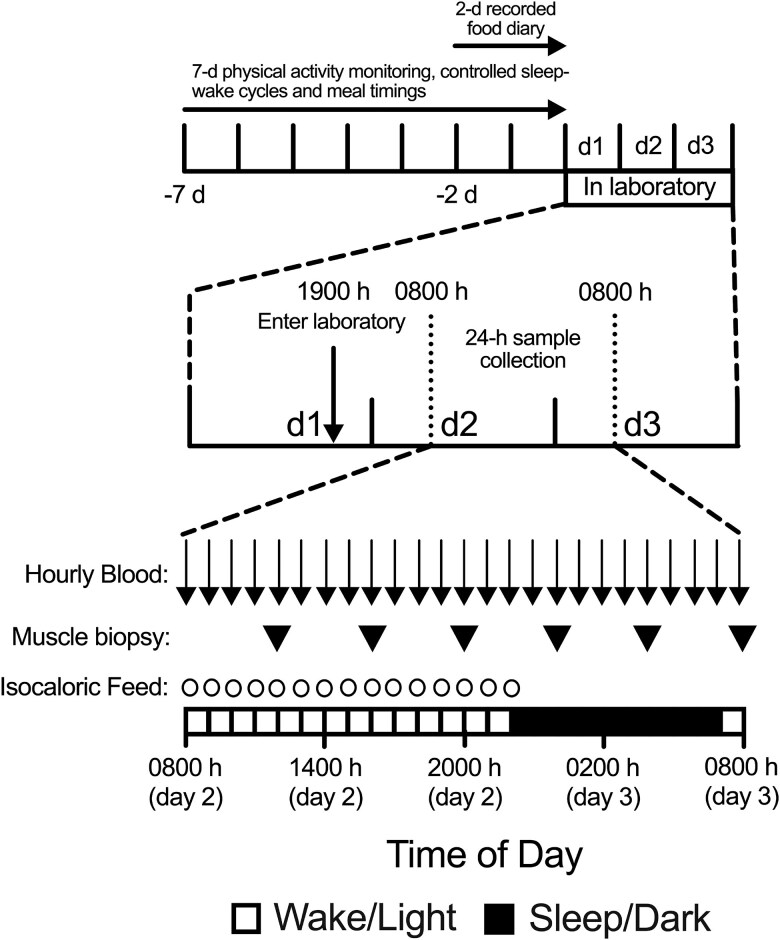
Schematic representation of the study protocol.

Following the standardization week, participants reported to the laboratory at 19:00 on experimental day 1 to acclimatize to the laboratory (Fig. 1). Laboratory conditions were standardized for the duration of their stay, with blackout blinds to prevent the penetration of natural light and room temperature maintained at 20 to 25 °C. During waking hours, artificial lighting was set at 800 lux in the direction of gaze (07:00–22:00 hours) and turned off (0 lux) during sleeping hours (22:00–07:00 hours), during which time participants wore an eye mask. Participants remained in a semi-recumbent position throughout (ie, head-end of bed elevated to 30^o^). Upon arrival, participants were shown to their bed and provided with a prescribed meal composed of a baked potato with butter and cheese, steamed vegetables (broccoli and mini corn), followed by a bowl of fresh strawberries, raspberries, and blueberries (1245 kcal; 31% carbohydrate, 50% fat, and 19% protein). An instant hot chocolate made with whole milk was then provided at 21:30 (242 kcal; 56% carbohydrate, 24% fat, and 20% protein) before lights out at 22:00 hours.

On day 2, participants were awakened at 07:00 hours and RMR was immediately measured over 15 minutes using indirect calorimetry via the Douglas bag technique (60). An intravenous cannula was inserted to an antecubital vein to allow for hourly 10-mL blood draws from 08:00 hours, alongside appetite visual analog scale (VAS) during waking hours (reported previously (50)). Muscle biopsies were collected every 4 hours from 12:00 hours on day 2 through to 08:00 hours on day 3. After each set of measurements, an hourly feed (commencing at 08:00 hours) was ingested in the form of a meal-replacement solution (1.25 kcal·mL^−1^, 45% carbohydrate, 25% fat, 30% protein; Resource Protein, Nestlé; Vevey, Switzerland). Each hourly dose was prescribed to give 6.66%·h^−1^ of measured 24-hour RMR across the 15-hour wake period (118 ± 19 kcal·h^−1^). Plain water was consumed ad libitum and participants had access to mobile devices, on-demand entertainment, music, and reading material throughout waking hours only. Toilet breaks were permitted in the first half of each hour as required.

The final set of waking measurements were collected at 22:00 hours, along with ingestion of the final prescribed feed. Following this, the lights were switched off and participants were asked to wear an eye mask throughout the lights-out period. Blood samples continued throughout the night at hourly intervals without intentionally waking the participants. Participants were awakened at 07:00 hours, and a blood sample was immediately drawn. The final set of measurements were made at 08:00 hours.

### Outcome Measures

#### Blood sampling and analysis

At each time point, 10 mL of whole blood was drawn and immediately distributed into tubes treated with lithium heparin (for analysis of melatonin) or EDTA (for analysis of glucose, insulin, nonesterified fatty acids, glycerol-corrected triglycerides, glycerol and C-terminal telopeptide) or left to clot at room temperature for 15 minutes (Serum; for analysis of cortisol and testosterone). Blood collection tubes were centrifuged for 10 minutes (3466*g*, 4 °C), after which the supernatants were removed and stored at −80 °C.

Plasma melatonin concentration was measured in the heparinized samples using a radioimmunoassay (Surrey Assays Ltd, UK; assay performance reported elsewhere (50)). Plasma insulin (Mercodia, Sweden; RRID: AB_2877672; intra-assay coefficient of variation [CV] 6%, inter-assay CV 13%); C-terminal telopeptide (CTX; Immunodiagnostic systems [IDS] UK; RRID: AB_2923399; intra-assay CV 19%, inter-assay CV 27%); glucose (intra-assay CV 3%, inter-assay CV 3%); nonesterified fatty acids (NEFA; intra-assay CV 6%, inter-assay CV 6%); glycerol (intra-assay CV 12%, inter-assay CV 18%); and triglycerides (intra-assay CV 4%, inter-assay CV: 18%) (Randox, UK) were quantified in EDTA-treated plasma, with cortisol (Tecan, CH; RRID: AB_2924715; intra-assay CV 6%, inter-assay CV 7%) and testosterone (R&D Systems, Bio-Techne, US; RRID: AB_2820244; intra-assay CV 30%, inter-assay CV 28%) quantified in serum.

#### Skeletal muscle sampling and analysis

Skeletal muscle samples were collected from the *vastus lateralis* under local anesthesia (1% lidocaine: Hameln Pharmaceuticals Ltd., Brockworth, UK). Samples were collected at 4-hourly intervals from 12:00 until 08:00 hours (ie, 6 in total) from a 3 to 5 mm incision in the anterior aspect of the thigh using a Bergstrom needle adapted for suction (61, 62). Samples were taken from each leg in a randomly determined alternating order between dominant and nondominant leg, ascending up the leg with skin incisions separated by 2 to 3 cm. Daytime biopsies were taken following the VAS and blood sample, but before the prescribed feed. Thirty minutes prior to sleep, incisions for the nighttime biopsies were made to minimize disruption to participants’ sleep. For nighttime tissue biopsies (ie, 00:00 and 04:00 hours), participants were awakened briefly but continued to wear the eye mask while samples were taken by torchlight (samples acquired and researchers left the laboratory within 3-5 minutes). Samples were immediately snap-frozen in liquid nitrogen for subsequent storage at −80°C.

Samples were later homogenized in 2 mL Trizol (Invitrogen, UK) and centrifuged 2500*g* for 5 minutes at 4°C. The top layer and pellet were removed and 200 μL of chloroform was added per 1 mL of Trizol and mixed vigorously for 15 seconds. Samples were subsequently incubated at room temperature for 3 minutes prior to centrifugation at 2500*g* for 5 minutes at 4 °C. The aqueous phase was then removed and mixed with an equal volume of 70% ethanol prior to loading on a RNeasy mini column for extraction (Qiagen, Crawley, UK). All samples were quantified using spectrophotometry, with 2 μg of total RNA reverse transcribed using a high-capacity cDNA reverse transcription kit (Applied Biosystems, Warrington, UK). Taqman low-density Custom Array using Micro Fluidic cards (Life Technologies, Thermo Fisher Scientific) was used for the relative quantification of expression of 45 genes listed in Table 3, as previously described (63, 64). The geometric mean of 18S ribosomal RNA (*18S*) (Hs03003631_g1), Actin alpha 1, skeletal muscle (*ACTA1*) (Hs05032285_s1), and hydroxymethylbilane synthase (*HMBS*) (Hs00609296_g1) was used as an endogenous control. The comparative threshold cycle (Ct) method was used to process the data where ΔCt = Ct target gene—Ct endogenous control (Geometric mean of 18 seconds, *Actin, HMBS*); cosinor analysis on the raw Ct values of *18S, ACTA1,* and *HMBS* did not indicate 24-hour rhythmicity across the protocol, with mean ± SD Ct values demonstrating high stability over all timepoints (10.2 ± 0.2, 15.4 ± 0.2 and 27.9 ± 0.1, respectively). Data were then normalized to an internal calibrator and finally 24-hour mean expression. One gene (*OTX1*; Orthodenticle Homeobox 1) was undetectable and, therefore, data for 44 genes are presented.

**Table 3.** Gene expression assay targets in human skeletal muscle (*vastus lateralis*)

Gene	Protein/enzyme	Assay ID
*18S rRNA*	18S ribosomal RNA	Hs03003631_g1
*ACTA1*	Actin alpha 1, skeletal muscle	Hs05032285_s1
*HMBS*	Hydroxymethylbilane synthase	Hs00609296_g1
*ARNTL*	Basic helix-loop-helix ARNT like 1	Hs00154147_m1
*CLOCK*	Circadian Locomotor Output Cycles Kaput	Hs00231857_m1
*CRY1*	Cryptochrome circadian regulator 1	Hs00172734_m1
*CRY2*	Cryptochrome circadian regulator 2	Hs00901393_m1
*CSN1KE*	Casein kinase 1 epsilon	Hs01095999_g1
*NPAS2*	Neuronal PAS domain protein 2	Hs00231212_m1
*NR1D1*	Nuclear receptor subfamily 1 group D member 1	Hs00253876_m1
*NR1D2*	Nuclear receptor subfamily 1 group D member 2	Hs00233309_m1
*PER1*	Period circadian protein 1	Hs00242988_m1
*PER2*	Period circadian protein 2	Hs01007553_m1
*PER3*	Period circadian protein 3	Hs00213466_m1
*TP53*	Tumor protein p53	Hs01034249_m1
*MYH1*	Myosin heavy chain 1	Hs00428600_m1
*MYOD1*	Myogenic differentiation 1	Hs00159528_m1
*FOXO3*	Forkhead box O3	Hs00818121_m1
*FBXO32*	F-box protein 32	Hs01041408_m1
*MTOR*	Mechanistic target of rapamycin kinase	Hs00234508_m1
*SIRT1*	Sirtuin 1	Hs01009006_m1
*AKT1*	AKT serine/threonine kinase 1	Hs00178289_m1
*B4GALT5*	beta-1,4-galactosyltransferase 5	Hs00941041_m1
*CS*	Citrate synthase	Hs02574374_s1
*HK2*	Hexokinase 2	Hs00606086_m1
*GLUT4*	Solute carrier family 2-member 4	Hs00168966_m1
*PDK4*	Pyruvate dehydrogenase kinase 4	Hs01037712_m1
*CPT1B*	Carnitine palmitoyltransferase 1B	Hs00189258_m1
*FABP3*	Fatty acid binding protein 3	Hs00997362_m1
*PPARD*	Peroxisome proliferator activated receptor delta	Hs04187066_g1
*PPARG*	Peroxisome proliferator activated receptor gamma	Hs00173304_m1
*PRKAA1*	Protein kinase AMP-activated catalytic subunit alpha 1	Hs01562315_m1
*PRKAA2*	Protein kinase AMP-activated catalytic subunit alpha 2	Hs00178903_m1
*ALAS1*	5′-aminolevulinate synthase 1	Hs00963537_m1
*CYCS*	Cytochrome c, somatic	Hs01588974_g1
*PPARGC1A*	PPARG coactivator 1 alpha	Hs00173304_m1
*SIRT3*	Sirtuin 3	Hs00953477_m1
*TFAM*	Transcription factor A, mitochondrial	Hs00273372_s1
*UCP3*	Uncoupling protein 3	Hs01106052_m1
*MAPK1*	Mitogen-activated protein kinase 1	Hs01046830_m1
*MAPK3*	Mitogen-activated protein kinase 3	Hs00385075_m1
*MAPK14*	Mitogen-activated protein kinase 14	Hs01051152_m1
*MAL*	Myelin and Lymphocyte T-cell differentiation protein	Hs00707014_s1
*CREB5*	cAMP responsive element binding protein 5	Hs00191719_m1
*EIF4EBP1*	Eukaryotic translation initiation factor 4E binding protein 1	Hs00607050_m1
*HNRNPDL*	Heterogeneous nuclear ribonucleoprotein D like	Hs00943609_m1
*RPS6*	Ribosomal protein S6	Hs04195024_g1

### Statistical Analysis

Concentrations for circulating metabolic and endocrine markers were adjusted to melatonin onset for each participant as determined by the 25% method (ie, calculation of when 25% of the peak melatonin concentration occurred) (65). The time in minutes between melatonin onset and midnight was calculated for each participant and used to adjust 24-hour profiles. The resulting x-values were binned around half past the hour with average y-values plotted at half past the hour (66-68). Muscle data were not adjusted for melatonin onset as 4-hourly sampling resolution was not deemed sufficient for this type of subtle adjustment.

Analysis of rhythmicity for all outcomes was conducted using the cosine method (Prism 9, Graphpad; CA, USA). In this approach, a cosine wave is fit to the 24-hour profile of a given variable and compared against a horizontal line through the mean values (null). If a cosine wave provides a better fit (*R^2^*) for the data than the horizontal line then the dataset characterizes diurnal (or 24-hour) rhythmicity, with the mesor (rhythm-adjusted mean), amplitude (magnitude of the difference between mesor and peak/trough values) and acrophase (timing of rhythmic peak) all identified and reported (56, 69). Reported *P *values are the output of the Extra sum-of-squares F test. This method was chosen a priori to provide a greater descriptive characterization of temporal patterns compared to commonly used statistical approaches such as analyses of variance (eg, 1-way ANOVA looking at effects of time or 2-way ANOVA for treatment × time interactions) but it must also be recognized that different analytical approaches may yield varied results (56). While post hoc adjustment of *P *values for multiple statistical tests is sometimes required to minimize inflation of type I error rates (ie, false positives), it has been questioned whether such adjustment is always necessary (70), and it is rare to see such adjustment between separate outcome measures. Moreover, given the aims of the study to characterize rhythmicity in metabolic outcomes, it was not deemed necessary to perform such adjustments. All data are presented as mean ± SD unless otherwise stated (eg, figures are mean ± 95% CI).

## Results

### Metabolites

**Figure 2.**
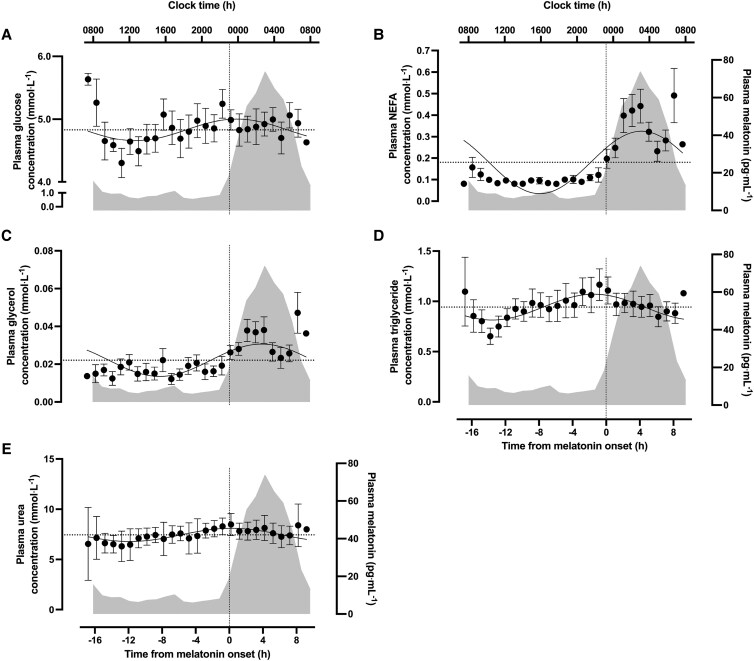
24-hour profile for melatonin onset adjusted (A) plasma glucose, (B) plasma NEFA, (C) plasma glycerol, (D) plasma triglycerides, and (E) plasma urea. Solid lines denote the regression that best fits the data with the horizontal dotted line representing the 24-hour mean concentration used for the null comparison. The dotted vertical line denotes melatonin onset. The shaded areas represent 24-hour melatonin profile.

All plasma metabolites displayed diurnal rhythmicity. Mean plasma glucose was rhythmic (*P* = .04, *R^2^* = 0.03, Fig. 2A). The acrophase occurred at 01:19 hours and fell to the nadir in the afternoon, with a mean concentration of 4.83 ± 0.44 mmol·L^−1^ and amplitude of 0.17 mmol·L^−1^. Plasma NEFA was also rhythmic, peaking at 04:56 hours and falling to the nadir in the afternoon, with an amplitude of 0.15 mmol·L^−1^ and rhythm-adjusted mean of 0.18 ± 0.05 mmol·L^−1^ (*P* < .01, *R^2^* = 0.38, Fig. 2B). Likewise, diurnal rhythmicity was evident in plasma glycerol. Mean concentrations across the period were 0.02 ± 0.01 mmol·L^−1^ and the diurnal rhythm was characterized by an amplitude of 0.08 mmol·L^−1^, peaking at 04:32 hours, with lowest values in the afternoon (*P* < .01, *R^2^* = 0.14, Fig. 2C). Plasma triglycerides were also rhythmic, with the acrophase occurring at 23:14 hours and falling to a nadir in the afternoon, with an amplitude of 0.13 mmol·L^−1^ and 24-hour mean of 0.94 ± 0.32 mmol·L^−1^ (*P* < .01, *R^2^* = 0.06, Fig. 2D). Finally, plasma urea was rhythmic across the period, peaking at 00:46 hours, with an amplitude of 0.66 mmol·L^−1^ and mean concentration of 7.45 mmol·L^−1^ (*P* < .01, *R^2^* = 0.08, Fig. 2E).

### Hormones and Telopeptides

**Figure 3.**
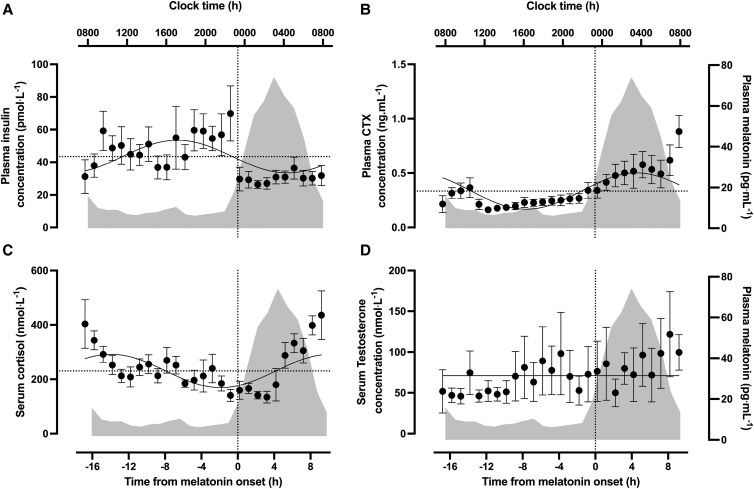
24-hour profile for melatonin onset adjusted (A) plasma insulin, (B) plasma C-terminal telopeptide (CTX), (C) serum cortisol, and (D) serum testosterone. Solid lines denote the regression that best fits the data with the horizontal dotted line representing the 24-hour mean concentration used for the null comparison. The dotted vertical line denotes melatonin onset. The shaded areas represent 24-hour melatonin profile.

Plasma insulin was rhythmic, peaking at 18:04 hours before falling to an overnight nadir (*P* < .0001, *R^2^* = 0.08, Fig. 3A). The diurnal rhythm occurred with an amplitude of 10.0 pmol·L^−1^ and a mean concentration of 43.4 ± 17.1 pmol·L^−1^. Plasma CTX was also characterized by diurnal rhythmicity (*P* < .0001, *R^2^* = 0.19, Fig. 3B); peak concentration occurred at 05:07 hours and fell to the nadir during the afternoon, with an amplitude of 0.16 ng·mL^−1^ and mean of 0.29 ± 0.20 ng·mL^−1^. Serum cortisol was also rhythmic, peaking at 10:50 hours with an amplitude of 22.3 nmol·L^−1^ (*P* < .0001, *R^2^* = 0.12, Fig. 3C). Average cortisol concentration across the 24-hour period was 232 ± 55 nmol·L^−1^. Conversely, mean serum testosterone was not rhythmic with an average concentration of 70.2 ± 54.8 nmol·L^−1^ (*P* = .62, Fig. 3D). Melatonin data are reported elsewhere (24, 50). Briefly, peak plasma melatonin occurred at 03:30 hours ± 55 minutes with and mean melatonin onset occurred at 23:18** **hours ± 46 minutes (Figs. 2 and 3). Correction of all outcomes to melatonin onset resulted in an average shift from clock time of 42 minutes (range, −35 to 117 minutes).

### Skeletal Muscle Gene Expression

**Figure 4.**
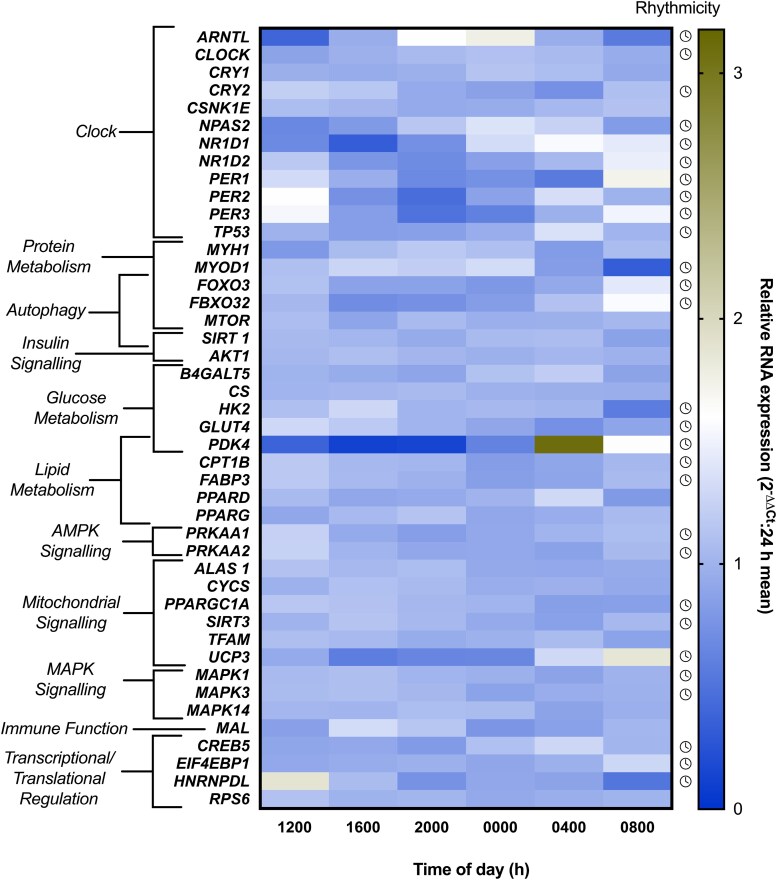
Relative changes in skeletal muscle RNA expression across the 24-hour semi-constant routine. Diurnal rhythmicity (as determined by cosinor analysis) is denoted by a clock symbol.

**Figure 5.**
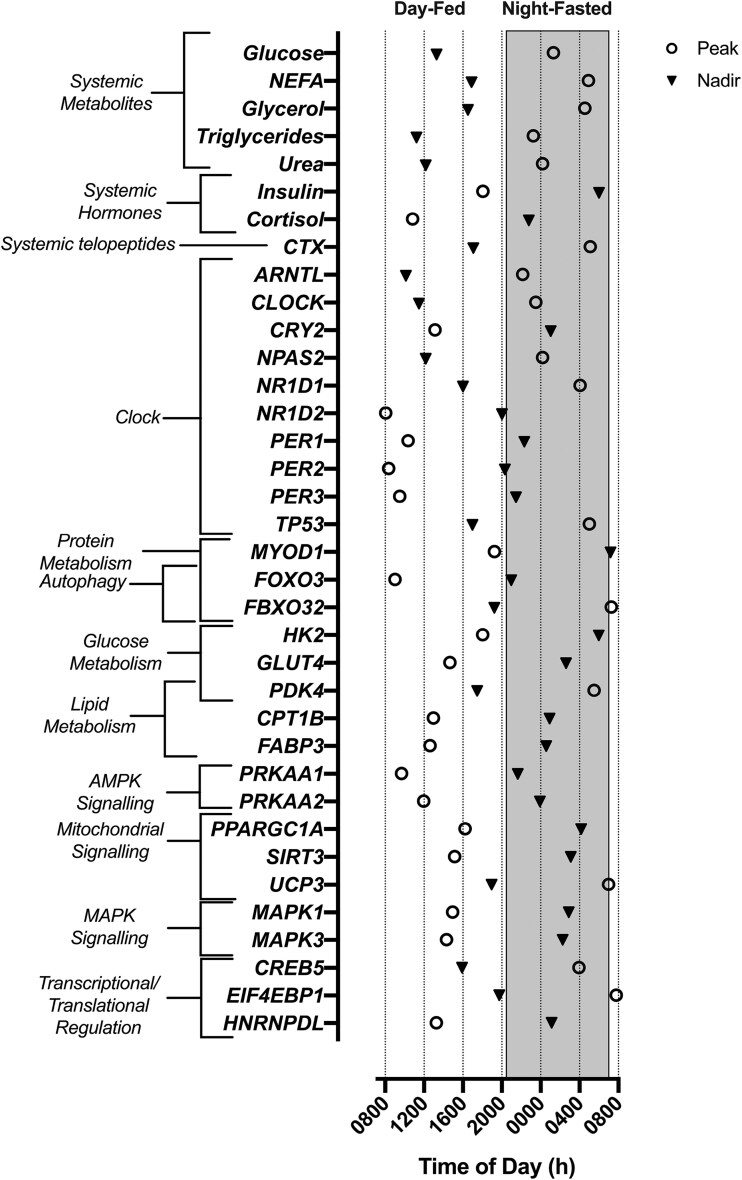
Peak (circles) and nadir (triangles) timings of circulating metabolites, hormones, telopeptides, and skeletal muscle genes displaying significant diurnal rhythmicity. The dark/fasted period is depicted in the shaded gray region.

Of the 44 genes assessed, 26 displayed rhythmicity (all *P* < .05) (Fig. 4). This diurnal rhythmicity was evident for core clock genes (acrophase, amplitude %): *ARNTL* (22:18 hours, 70%)*, CLOCK* (23:29 hours, 11%)*, CRY2* (13:08 hours, 23%)*, NPAS2* (00:12 hours, 37%)*, NR1D1* (04:04 hours, 63%)*, NR1D2* (08:04 hours, 36%)*, PER1* (10:21 hour, 48%)*, PER2* (08:21 hour, 41%)*, PER3* (09:30 hours, 57%), and *TP53* (05:00 hours, 20%). Genes relating to autophagy and protein metabolism were also rhythmic: *MYOD1* (19:14 hours, 41%), *FOXO3* (09:00 hours, 26%), *FBXO32* (07:16 hours, 39%). Diurnal oscillations were also present in the expression of genes involved in glucose and lipid metabolism; *GLUT4* (14:40 hours, 25%), HK2 (18:28 hours, 21%), *FABP3* (12:37 hours, 15%)*, PDK4* (05:30 hours, 133%), and *CPT1B* (12:58 hours, 14%). Finally, diurnal variation was apparent in genes involved in mitochondrial signaling; *PPARGC1A* (16:13 hours, 15%) and *UCP3* (06:59 hours, 58%), *SIRT3* (15:09 hours, 10%) as well as transcriptional/translational regulation and MAPK signaling; *CREB5* (03:57 hours, 19%)*, EIF4EBP1* (07:41 hour, 11%), and *HNRNPDL* (13:17 hours, 35%). Temporal relationships between rhythmic circulating biomarkers and skeletal muscle genes are reported in Fig. 5.

## Discussion

This is the first study to report serial measures of human skeletal muscle alongside systemic markers of metabolic regulation under controlled diurnal conditions. Diurnal rhythmicity was apparent in skeletal muscle genes relating to carbohydrate, lipid and protein metabolism, autophagy, and mitochondrial signaling as well as in circulating glucose, insulin, NEFA, glycerol, triglycerides, cortisol, and CTX.

Plasma insulin was rhythmic, peaking in the evening (∼18:00 hours) and falling to nadir overnight (∼04:00 hours). This is consistent with previous research employing a continuous glucose clamp (71) and generally agrees with the notion of greater insulin sensitivity in the morning compared to the evening (11). However, the timing of peak insulin differs from that reported in Wehrens et al (72), in which the acrophase of insulin occurred ∼8 to 11 hours after a melatonin onset similar to that reported currently, placing peak time at ∼07:00 to 10:00 hours. Nevertheless, methodological differences between studies allow for greater understanding of behavioral factors that may influence such rhythms. The continuous feeding pattern during waking hours in the current study suggests rhythmicity in circulating insulin occurs at least partly independent of food intake (73-75). Nonetheless, insulin is highly responsive to nutrient intake, and the coincidence of the overnight fast with lower nocturnal insulin suggests nutrient intake could be producing some of the apparent diurnal responses. Plasma glucose concentrations were also rhythmic (peak ∼01:30 hours), consistent with studies of circadian misalignment, constant routine, and forced desynchrony thus further highlighting robust regulation of rhythms in plasma glucose by the endogenous clock even under controlled diurnal conditions (2, 3, 5, 76). Interestingly, while glucose and insulin concentrations might usually be expected to correlate when comparing acute meal responses over the minutes following feeding, the current model of hourly feeding and sampling over 24 hours may explain why variance in insulin may be sufficient to alter glucose kinetics/flux but without necessarily being reflected by changes in the systemic concentrations of glucose. At the tissue level, skeletal muscle *GLUT4* and *PPARGC1a* RNA were rhythmic, with peak levels occurring at ∼15:00 and ∼16:00 hours, respectively (ie, when insulin was rising), with the lowest levels at ∼04:00 hours (ie, when insulin was lowest). Peak *HK2* RNA occurred at ∼18:30 hours, shortly after the rhythmic peak in plasma insulin and therefore in line with the regulatory effects of insulin on hexokinase activity (77, 78). The observation of rhythms in the skeletal muscle expression of *GLUT4* and *HK2* is contrary to previous work in mice whereby no significant oscillations in these genes (79, 80). Collectively, the broad alignment of the rhythms of these genes with rhythmic plasma insulin reflects their involvement in skeletal muscle glucose uptake and their potential to influence diurnal glucose metabolism (81, 82).

The diurnal profiles of NEFA and glycerol were also broadly anti-phasic to the 24-hour profile of insulin (Fig. 5). Circulating NEFA and glycerol were generally suppressed during waking hours, before rising to peak at ∼04:00 to 05:00 hours, consistent with the nocturnal rise reported in previous literature (83-85). Plasma triglycerides were also rhythmic under controlled diurnal conditions, whereby systemic concentrations were low during the morning before rising to a peak at ∼23:30 hours (Fig. 2D). The rhythmic profile of these circulating lipids is consistent with the regulatory effects of insulin on adipose tissue lipolysis (86-88) and circulating triglyceride levels (89). The anti-phasic relationship between insulin with NEFA and glycerol alongside the aligned rhythms in insulin and triglycerides could be reflective of feeding status and the subsequent changes in adipose tissue lipolysis in the overnight fasted state (45, 46, 49). Circulating melatonin is speculated to in part contribute toward the regulation of lipid metabolism (90, 91), this may be reflected in the temporal similarity in acrophase among systemic melatonin NEFA and glycerol (Fig. 5); however, further work is required to better understand the effects of melatonin on lipid metabolism.

Peak expression of skeletal muscle *PDK4* RNA (∼05:30 hours) occurred proximally to the peak in systemic NEFA (Fig. 5). This is consistent with previous work demonstrating an association between diurnal variation in *PDK4* and NEFA, which may be explained by the role of this gene in stimulating fatty acid utilization in response to a rise in NEFA availability (92-96). This temporal pattern may be driven by the diurnal feeding pattern present in both the current and previous work (96). However, following peak RNA levels, *PDK4* declined at ∼08:00 hours, despite the continual fasted state and resultant elevated NEFA availability, suggesting observed effects may not be solely due to the imposed feeding pattern. The profile of genes involved in the regulation of solubility, mobility, and transport of fatty acids (eg, *CPT1B* and *FABP3*) did not align with systemic concentrations of NEFA (97, 98), but broadly mirrored the rhythm in insulin. Furthermore, alignment between *UCP3* expression with the profile of systemic NEFA is consistent with the involvement of this gene in mitochondrial fatty acid oxidation (99, 100).

Plasma urea concentration increased gradually through waking hours (peak ∼00:46 hours), before declining overnight. This could be in response to the imposed feeding pattern, reflecting a greater rate of nitrogen excretion later in the day once the total amount of nutrients had been consumed and subsequent decrease in response to the withdrawal of nutrition during sleep (101, 102).

Numerous metabolic and endocrine responses relevant to tissue turnover show diurnal rhythms under semi-constant routine. Cortisol displayed the expected rhythm, peaking at (∼11:00 hours) before falling to its lowest value in the evening, approximately coinciding with melatonin onset (72). Peak expression of skeletal muscle *FBXO32* occurred during the morning period while cortisol was rising; this is consistent with the related action of this gene and hormone in catabolic processes, which may be driven by the diurnal overnight fast (103-107). Following muscle breakdown, autophagy is a vital process to stimulate muscle regeneration (39). Expression of *FOXO3*, which promotes expression of downstream targeted autophagy-related proteins, also peaked in the morning when cortisol is rising, which may reflect the proposed regulatory effects of cortisol in stimulating increased autophagic flux in skeletal muscle (108, 109). Collectively the temporal patterns of these skeletal muscle genes hint at diurnal fluctuations in tissue turnover, which has previously been observed in nonhuman models (107, 110). However, serum testosterone did not display diurnal rhythmicity. Previous studies have demonstrated rhythmicity in systemic testosterone, with highest values early in the morning (∼08:00 hours) and corresponding lowest values ∼12 hours later (18, 111-113). This typical rhythm was not observed in the current study, which could be explained by several mechanisms, including daytime hourly nutrition (114, 115), sleep fragmentation (116), and the potential acute effect of muscle biopsies on systemic cortisol (117). The lack of rhythmicity could also be due to the sensitivity of measurement through the use of commercial enzyme-based immuno-assays rather than gold standard measurement by liquid chromatography–mass spectrometry (118, 119). Equally, neither free testosterone nor sex hormone–binding globulin were assessed as part of these analyses, both of which have been reported to display clear daily rhythms (113, 120). Finally, *MYOD1*, an important myogenic regulatory factor, displayed a similar peak and nadir to insulin. This is in line with the proposed effects of insulin on muscle protein turnover, hinting at diurnal patterns in skeletal muscle turnover, which are plausibly driven by patterns of feeding and fasting (77, 121).

Plasma CTX was lowest during the day in the fed state and peaked during the biological night in the fasted state (∼05:00 hours) in a remarkably similar rhythm and amplitude to previous literature (122-124). Feeding reliably suppresses bone resorption, and acute fasting dampens typical rhythmicity (123). The current data therefore highlight the influence of diurnal feeding-fasting cycles on the typical 24 hours patterns of systemic CTX (125, 126). However, plasma CTX was higher at the end of the measurement period than the beginning, suggesting that other factors, such as sleep and wake cycles, may also impact bone resorption and future work should seek to establish the contribution of sleep on bone resorption independent of nutritional status (127, 128).

Despite the novelty of simultaneously collected plasma and muscle samples under controlled diurnal conditions in a 24-hour period, the current data must be interpreted in light of several factors. Participants were fed relative to individualized requirements, to account for the role of RMR as a driver of energy intake and appetite (53, 54). However, 24-hour bed rest eliminates the influence of physical activity on circadian clocks, glucose, lipid, and protein metabolism in skeletal muscle as well as bone turnover (42, 129-131). This is especially pertinent given that muscle samples were collected from the legs, which typically sustain greater load bearing than upper limbs, so bed rest may elicit greater metabolic perturbation (132). The potential for multiple tissue biopsies on localized inflammation must also be acknowledged. However, biopsies were taken from alternating limbs with each following biopsy on the same limb being taken 3 cm proximally to the initial incision. This is in line with Van Thienin and colleagues (133), who reported inflammatory markers were upregulated at the distal, but not at the proximal site when taking sequential samples from the same limb. Equally, it is a limitation of this study that sleep duration and quality were not objectively measured, so it is not possible to comment on the impact of nocturnal sampling on those outcomes or their potential influence on the primary outcomes. It should also be considered that the bright light in the laboratory may have delayed the melatonin onset time and therefore suppressed the release of melatonin in the first part of the night (134).

The use of a “semi-constant” routine with alignment of the dark-light cycle with fasting/food intake and sleep/wakefulness can be viewed as both a strength and a limitation of the current study. The model has ecological validity since the semi-constant routine reflects free-living environmental and behavioral cycles that exist outside of the laboratory; however, the presence of such diurnal factors also makes it more difficult to disentangle whether rhythms are truly circadian or driven by behavioral/environmental cycles.

Despite the aforementioned factors, diurnal rhythmicity was still observed in the majority of core clock genes, highlighting the robust rhythmic nature of skeletal muscle (25). While the current findings hint at the possibility of diurnal influences of feeding patterns on circulating and tissue rhythms, direct comparison of divergent nutrient feeding patterns, especially where nutrition is provided through the night, is required to establish whether the observed rhythms are driven endogenously or by the imposed behavioral (feeding and sleep) factors (135).

In summary, this was the first study to measure diurnal rhythms in human skeletal muscle gene expression alongside systemic metabolites and hormones under controlled diurnal conditions. The diurnal pattern in genes relating to carbohydrate and lipid metabolism tended to reflect the pattern of insulin across 24 hours, which may in part be driven by the diurnal influence of cyclic feeding and fasting. This study provides novel context for metabolic regulation at both the tissue and systemic level.

## Data Availability

Some or all datasets generated during and/or analyzed during the current study are not publicly available but are available from the corresponding author on reasonable request.

## Abbreviations

CTX: C-terminal telopeptide
CV: coefficient of variation
NEFA: nonesterified fatty acids
RMR: resting metabolic rate
VAS: visual analog scale
